# Polymorphisms of the Tissue Inhibitor of Metalloproteinase 3 Gene Are Associated with Resistance to High-Altitude Pulmonary Edema (HAPE) in a Japanese Population: A Case Control Study Using Polymorphic Microsatellite Markers

**DOI:** 10.1371/journal.pone.0071993

**Published:** 2013-08-22

**Authors:** Nobumitsu Kobayashi, Masayuki Hanaoka, Yunden Droma, Michiko Ito, Yoshihiko Katsuyama, Keishi Kubo, Masao Ota

**Affiliations:** 1 First Department of Internal Medicine, Shinshu University School of Medicine, Matsumoto, Nagano, Japan; 2 Department of Legal Medicine, Shinshu University School of Medicine, Matsumoto, Nagano, Japan; 3 Department of Pharmacy, Shinshu University Hospital, Matsumoto, Nagano, Japan; St. Petersburg Pasteur Institute, Russian Federation

## Abstract

**Introduction:**

High-altitude pulmonary edema (HAPE) is a hypoxia-induced, life-threatening, high permeability type of edema attributable to pulmonary capillary stress failure. Genome-wide association analysis is necessary to better understand how genetics influence the outcome of HAPE.

**Materials and Methods:**

DNA samples were collected from 53 subjects susceptible to HAPE (HAPE-s) and 67 elite Alpinists resistant to HAPE (HAPE-r). The genome scan was carried out using 400 polymorphic microsatellite markers throughout the whole genome in all subjects. In addition, six single nucleotide polymorphisms (SNPs) of the gene encoding the tissue inhibitor of metalloproteinase 3 (*TIMP3*) were genotyped by Taqman® SNP Genotyping Assays.

**Results:**

The results were analyzed using case-control comparisons. Whole genome scanning revealed that allele frequencies in nine markers were statistically different between HAPE-s and HAPE-r subjects. The SNP genotyping of the *TIMP3* gene revealed that the derived allele C of rs130293 was associated with resistance to HAPE [odds ratio (OR) = 0.21, P = 0.0012) and recessive inheritance of the phenotype of HAPE-s (P = 0.0012). A haplotype CAC carrying allele C of rs130293 was associated with resistance to HAPE.

**Discussion:**

This genome-wide association study revealed several novel candidate genes associated with susceptibility or resistance to HAPE in a Japanese population. Among those, the minor allele C of rs130293 (C/T) in the *TIMP3* gene was linked to resistance to HAPE; while, the ancestral allele T was associated with susceptibility to HAPE.

## Introduction

High-altitude pulmonary edema (HAPE) is a hypoxia-induced, non-cardiogenic pulmonary edema characterized by exaggerated pulmonary hypertension, which leads to pulmonary capillary stress failure and causes a high permeability type of life-threatening pulmonary edema [1], [2]. HAPE occurs in healthy and often young individuals within 2–4 days after rapid exposure to high altitudes over 2,500 meters above sea level (m), and lack of oxygen is the crucial cause of this disease [1], [2]. Although the mechanisms underlying the pathogenesis of HAPE are complex, the generally proposed paradigm is a sequential process of hypoxia-induced pulmonary hypertension, increased permeability of pulmonary capillaries because of stress failure, and destruction of the alveolar epithelial membrane barrier, resulting in high permeability pulmonary edema [3], [4].

Some but not all affected individuals develop HAPE while exposed to high altitudes, probably due to individual variations in response to hypoxia. It was widely demonstrated that HAPE frequently occurs in individuals exposed to high altitudes [4]–[6], and several genetic studies have demonstrated that a genetic susceptibility may play a role in the development of HAPE [7], [8]. Genetic factors, including polymorphisms of the genes of nitric oxide synthase 3 (*NOS3*) [9], angiotensin-converting enzyme (*ACE*) [10], angiotensin II receptor (*AGTR1*) [10], and human leukocyte antigen *(HLA)* [11] have been positively associated with HAPE susceptibility in Japanese subjects. However, these candidate genetic factors were identified by genetic variant analysis in limited regions of the genome. A more comprehensive understanding of how genetic background influences the outcome of HAPE requires genome-wide association analyses.

The recently developed whole genome-wide association study can theoretically examine the entire genome in an unbiased fashion. This approach has been successfully applied to elucidate the genetic background underlying high-altitude indigenous populations and identified numerous important genes associated with hypoxia-tolerance in high-altitude populations [12]–[13]. However, the whole genome-wide association study has not yet been applied in studies evaluating genetic associations with susceptibility or resistance to HAPE. Thus, the present case-control association study, using 400 polymorphic microsatellite markers distributed throughout the whole genome, was performed in an attempt to identify the locations of candidate genes that might be associated with susceptibility or resistance to HAPE in a Japanese population. Association analysis using microsatellite markers is a powerful, yet cost-efficient method for mapping candidate susceptibility genes in multifactorial genetic diseases [14]. The HAPE susceptibility/resistance associations identified preferable genes near the significant microsatellite markers, which were further evaluated in a case-control association study using SNPs.

## Methods

### Ethics Statement

The current study was approved by the Ethics Committee of Shinshu University School of Medicine (Matsumoto, Japan). The protocol of the investigation was in accordance with the principals outlined in the Declaration on Helsinki of the World Medical Association and was approved by the Ethics Committee of Shinshu University School of Medicine. Written informed consent was obtained from each subject after a full explanation of the study.

### Subjects

We collected venous blood samples from 53 subjects susceptible to HAPE (HAPE-s) and 67 elite Alpinists resistant to HAPE (HAPE-r). All subjects were unrelated natives born in Japan and resided at low altitudes less than 610 m.

The 53 HAPE-s subjects were the patients of HAPE who were admitted to Shinshu University Hospital because of HAPE occurring during climbing in the Japan Alps at heights over 2,700 m from 1971 to 2009. The venous blood samples were collected and frozen under minus 70 Celsius degree for research purpose. The Shinshu University Hospital is the central facility for patients with HAPE in Matsumoto, a city located in the central part of Japan at a height of 610 m above sea level and surrounded by mountains of Japan Alps. The HAPE-s subjects consisted of 46 males and 7 females, ranging in age from 15 to75 years with an average age of 34.2 years. The diagnosis of HAPE was based on diagnostic criteria [15] at the onset of the disorder, and the differential diagnosis of acute mountain sickness (AMS) was made by computer tomography (CT) examination. All patients with HAPE recovered promptly within one week of hospitalization in Shinshu University Hospital. Related clinical examinations and cardiovascular tests were conducted in-hospital after recovery to exclude any preexisting cardiopulmonary problems.

The HAPE-r subjects consisted of 58 males and 9 females, ranging in age from 18 to 65 years with an average age of 37.0 years. They were elite mountaineers from the Mountaineering Association of Nagano Prefecture and the Alpine Club of Shinshu University and often climbed mountains higher than 3,000 m. They were invited to cooperate with our study for providing voluntarily their contributions (such as 7 ml venous blood) after a full explanation of the study. No subject reported any history of medical problems related to altitudes or cardiopulmonary disorders in a questionnaire, which contained the components of the Lake Louise Score [16], during recruitment. We defined these subjects as HAPE-r due to their resistance to HAPE during exposure to high-altitude environments.

### Preparation of Genomic DNA

Genomic DNA samples were extracted from all subjects from venous blood by phenol extraction of sodium dodecyl sulfate (SDS) - lysed and proteinase K-treated cells as described previously [9].

### Microsatellite Typing

Microsatellites are tandem arrays of short stretches of non-coding nucleotide sequences that usually repeat between 15–30 times [17]. They are usually used as molecular markers in the field of genetics. The obvious advantages of microsatellites are that heterozygosity is relatively high throughout the genome and that the typing can be generally performed by polymerase chain reaction (PCR) [14]. The genome scan was carried out using 400 polymorphic microsatellite markers with a resolution of 10.8 centiMorgan (cM) and average heterozygosity of 79% throughout the whole genome (http://www3.appliedbiosystems.com/cms/groups/mcb_marketing/documents/generaldocuments/cms_039831.pdf). Fluorescent-tagged (FAM, VIC, and NED) primers (ABI Linkage Mapping Set v.2.5-MD10) were purchased from Applied Biosystems, Foster City, CA, USA. The markers were amplified by PCR according to the manufacturer’s protocols in 10 µl reaction mixtures containing 40 ng genomic DNA. The PCR-amplified products were sequenced using GeneScan software and the fragment sizes were analyzed using an ABI 3130 DNA Analyzer. Semi-automated genotyping was performed using GeneMapper v3.5 (Applied Biosystems).

### Single Nucleotide Polymorphism (SNP) Genotyping

One marker, D22S280, located on chromosome 22q12.13 had a significant association with HAPE [corrected P (Pc) = 0.020] as shown in Table 1. We then used the National Center for Biotechnology Information (NCBI) Map Viewer (http://www.ncbi.nlm.nih.gov/mapview/) to select the gene encoding the tissue inhibitor of metalloproteinase 3 (*TIMP3*) as the most highly possible candidate gene and performed further genotyping with the SNPs of *TIMP3* in the HAPE-s and HAPE-r subjects (Table 2). The D22S280 marker is located within intron 1 of the *TIMP3* gene.

**Table 1:** The microsatellite markers with statistically significant associations with HAPE.

Markers	Cytobands	Code	HAPE-s	HAPE-r				
	on	of	N = 106	N = 134	OR	χ^2^	P*	Pc^†^
	Chromosomes	alleles	n	n				
D1S2697	1p36.13	284	11 (0.104)	2 (0.015)	7.64	9.119	0.0025	0.013
D1S230	1p31.3	156	18 (0.170)	46 (0.343)	0.39	9.107	0.0025	0.020
D5S424	5q13.3	212	47 (0.443)	36 (0.269)	2.17	7.988	0.0047	0.030
D6S257	6q12.1	179	26 (0.245)	13 (0.097)	3.03	9.560	0.0020	0.030
D12S368	12q13.13	202	43 (0.406)	81 (0.604)	0.45	9.368	0.0020	0.015
D14S283	14q11.2	139	24 (0.226)	54 (0.403)	0.43	8.411	0.0037	0.045
D16S3103	16p12.3	323	10 (0.094)	0 (0.0)	29.27	13.191	0.0003	0.003
D21S263	21q22.11	216	7 (0.066)	0 (0.0)	20.28	9.115	0.0030	0.035
D22S280	22q12.3	221	9 (0.087)	32 (0.239)	0.30	9.521	0.0020	0.020
D22S0112i‡	22q12.3	229	7 (0.066)	28 (0.209)	0.27	9.704	0.0018	0.018

Allele frequencies were expressed as decimals. N = total number of chromosomes; n = number of observed alleles.

HAPE = high-altitude pulmonary edema; HAPE-s = subjects susceptible to HAPE; HAPE-r = subjects resistant to HAPE.

* P values were calculated by Chi-square test (2×2 contingency table) for each allele.

Pc^†^ = corrected P, which was calculated by multiplying by the number of alleles in the given locus.

‡ D22S0112i: An additional marker located 19 bp centromeric region from D22S280.

**Table 2:** Candidate genes located around 100 kb from each significant marker shown in Table 1 *.

Chr.	Markers		Symbols	Descriptions
1		D1S2697†	*SPEN*	Spen homolog, transcriptional regulator
			*ZBTB17*	Zinc finger and BTB domain containing 17
			*C1orf64*	Chromosome 1 open reading frame 64
			*HSPB7*	Heat shock 27 kDa protein family, member 7
			*CLCNKA*	Chloride channel, voltage-sensitive Ka
			*CLCNKB*	Chloride channel, voltage-sensitive Kb
1		D1S230	*INADL*	InaD-like
5		D5S424†	*F2R*	Coagulation factor II (thrombin) receptor
			*F2RL1*	Coagulation factor II (thrombin) receptor like-1
			*S100Z*	S100 calcium binding protein Z
			*CHRBP*	Corticotropin releasing hormone binding protein
6		D6S257†	*COL21A1*	Collagen, type XXI, alpha1
12		D12S368	*KRT*	Keratin gene
14		D14S283	*TRAV*	T cell receptor alpha variable gene
16		D16S3103†	*XYLT1*	Xylosyltransferase I
21		D21S263†	*KRTAP*	Keratin associated protein gene
22		D22S280	*TIMP3*	Tissue inhibitor of mettaloproteinase 3
			*SYN3*	Synapsin III

Chr. = chromosome.

* Source: NCBI Map Viewer (http://www.ncbi.nlm.nih.gov/mapview/).

† Significant markers associated with HAPE susceptibility, otherwise associated with HAPE resistance.

Six SNPs distributed within *TIMP3* were used for genotyping. The selection criteria for the SNPs were based on the following information from the NCBI dbSNP database (build 37.3, http://www.ncbi.nlm.nih.gov/projects/SNP/), the HapMap database (http://hapmap.ncbi.nlm.nih.gov/downloads/index.html.en), and the SNP database of Applied Biosystems (http://bioinfo.appliedbiosystems.com/genome-database/snp-genotyping.html): (a) location within the *TIMP3* gene; (b) minor allele frequency over 10% in Japanese populations; (c) average heterozygosity of 30%; (d) density of at least one SNP per 5 kb; and (e) availability for validation assays. All six SNPs were genotyped using the Taqman® SNP Genotyping Assays (Applied Biosystems, Foster City, CA) using the Applied Biosystems 7500 Real-time PCR system according to manufacturer’s instructions.

### Statistical Analysis

Frequencies of alleles in each microsatellite marker were estimated by direct counting. The frequency was expressed as a decimal. The Hardy-Weinberg proportion (HWP) for multiple alleles was calculated by the Markov chain method within the GENPOP software package [18]. The Markov chain method has the advantage of obtaining a complete enumeration for testing Hardy –Weinberg equilibrium (HWE) in cases where the number of alleles and the sample size are small. The significant differences in allele frequencies between HAPE-s and HAPE-r were examined by the Chi-square test (2*×*2 contingency table). Fisher’s exact probability test was used instead of the Chi-square test for the comparisons when the number of subjects was less than 5. In the ABI Linkage Mapping Set v2.5, one marker contains several alleles those can be identified by allele size range in base pair (bp) observed using the ABI 3130 DNA Analyzer. Thus, a corrected P-value (Pc) was necessary for each marker in order to avoid false-positive statistical analysis. The Pc was calculated by multiplying the number of different alleles observed in each marker. Similarly, the Chi-square test (2*×*2 contingency table) was also applied for the comparisons of allele frequencies of the six SNPs in the *TIMP3* gene between HAPE-s and HAPE-r subjects. The strength of the associations with HAPE-s was estimated by odds ratios (OR) that were calculated as the cross-product ratio of a particular allele in the HAPE-s group compared with that in the HAPE-r group. An approximate 95% confidence interval (CI) of the odds ratio was given. Additionally, effects of the ancestral allele were calculated, assuming dominant as well as recessive modes of inheritance of the HAPE-s phenotype. The values (D’) of pair-wise linkage disequilibrium (LD) of the six SNPs were measured with Haploview software [19], which was then partitioned into block structures using common approaches of block definition, such as the Solid Spine of LD [20]. P and Pc values less than 0.05 indicated statistical significance.

## Results

The searching using four hundred polymorphic microsatellite markers revealed that five markers were associated with susceptibility to HAPE (defined by an OR greater than 2), namely D1S2697 (OR = 7.64, Pc = 0.013) on chromosome (chr) 1, D5S424 (OR = 2.17, Pc = 0.030) on chr 5, D6S257 (OR = 3.03, Pc = 0.030) on chr 6, D16S3103 (OR = 29.27, Pc = 0.003) on chr 16, and D21S263 (OR = 20.28, Pc = 0.035) on chr 21. In addition, four markers were associated with resistance to HAPE (defined by an OR smaller than 0.5), namely, D1S230 (OR = 0.39, Pc = 0.020) on chr 1, D12S368 (OR = 0.45, Pc = 0.015) on chr 12, D14S283 (OR = 0.43, Pc = 0.045) on chr 14, and D22S280 (OR = 0.30, Pc = 0.020) on chr 22 (Table 1). The cytoband positions of these significant markers on corresponding chromosomes are precisely provided in Table 1. The candidate genes within 100 kb of these significant markers presumed to be associated with susceptibility or resistance to HAPE are listed in Table 2 according to data from NCBI Map Viewer (http://www.ncbi.nlm.nih.gov/mapview/).

As shown in Table 1, among all the significant microsatellite markers associated with the susceptibility to HAPE, the D1S2697, D16S3103 and D21S263 markers showed the strongest associations (OR = 7.64, 29.27, and 20.28, respectively). However, the availability for validation was predicted to be very low for these markers because the HAPE-r group had none or few alleles in these markers. Therefore, we chose D22S280 as a marker of interest for further analysis as it showed the strongest association with HAPE (OR = 0.30, Pc = 0.020) among the rest of the significant markers (Table 1). To confirm the validation of this marker in association with HAPE in Japanese subjects, we selected an additional marker D22S0112i, which is located 19 bp centromeric region from D22S280, to verify the availability. We found allele 229 of D22S0112i had a significant association with HAPE (OR = 0.27, Pc = 0.018, Table 1). D22S0112i was extracted from the Gene Diversity Database System, Japan Biological Informatics Consortium (http://jbirc.jbic.or.jp/gdbs/top.jsp).

We next used the NCBI Map Viewer (http://www.ncbi.nlm.nih.gov/mapview/) to predict novel candidate genes within approximately 100 kb of the D22S280 marker, presuming an association with HAPE (Table 2). This revealed that the D22S280 marker (chromosome position: 33209428–33209626) was located in the *TIMP3* gene (chromosome position: 33196802–33259028).

The *TIMP3* gene was then supposed to be the most promising gene in associations with HAPE. The TIMP protein plays a crucial role in the physiological turnover of the extracellular matrix (ECM) by regulating matrix metalloproteinase (MMP) activities, which are strongly involved in the major pathophysiological phenotypes in lungs [21]. The *TIMP3* gene is expressed in many tissues including lungs [21]. Therefore, SNPs in the *TIMP3* gene were investigated and compared between the two groups.

All the examined SNPs in the *TIMP3* gene were in HWE for both HAPE-s and HAPE-r subjects. The SNP rs130293 was statistically significantly associated with HAPE-s (P = 0.00049, Table 3). The frequency of the derived allele C was significantly lower in HAPE-s subjects than HAPE-r subjects (P = 0.00049), indicating an association of the C allele with resistance to HAPE (OR = 0.22 with 95%CI from 0.09 to 0.55). In addition, the C allele was associated with recessive inheritance of the phenotype of HAPE-s (P = 0.0012, Table 3), probably due to a significantly lower frequency of the T/C heterozygous genotype in the HAPE-s group than the HAPE-r group (OR = 0.113 vs. 0.313, P = 0.009). None of the other five examined SNPs in the *TIMP3* gene were significantly associated with HAPE-s in the Japanese population (Table 3).

**Table 3:** The allele frequencies and genotype distributions of the six SNPs of the *TIMP3* gene in HAPE-s and HAPE-r subjects.

dbSNPs	Alleles*	Allele 1		OR	*P* †	Genotype distributions						*P* ‡	*P* §
		Frequency		(95% CI)		11		12		22		11/	12+22
	(1/2)	HAPE-s	HAPE-r			HAPE-s	HAPE-r	HAPE-s	HAPE-r	HAPE-s	HAPE-r	12+22	22
rs738992	C/T	0.481	0.515	0.87	0.60	0.192	0.328	0.577	0.373	0.231	0.299	0.0969	0.4084
				(0.52–1.46)									
rs130287	A/G	0.736	0.776	0.84	0.47	0.509	0.611	0.453	0.328	0.038	0.060	0.2605	0.5835
				(0.44–1.45)									
rs130293	C/T	0.057	0.216	0.22	0.00049	0	0.060	0.113	0.313||	0.887	0.627	0.0704	0.0012#
				(0.09–0.55)									
rs715572	G/A	0.721	0.746	0.88	0.66	0.519	0.567	0.404	0.358	0.077	0.075	0.6024	0.9625
				(0.49–1.57)									
rs2071947	C/T	0.577	0.627	0.81	0.43	0.327	0.373	0.500	0.507	0.173	0.119	0.6008	0.4066
				(0.48–1.37)									
rs9862	C/T	0.660	0.627	0.98	0.95	0.423	0.463	0.500	0.403	0.096	0.134	0.6021	0.4980
				(0.57–1.68)									

Allele frequencies and genotype distributions were expressed as decimals. SNPs = single nucleotide polymorphisms; HAPE-s = subjects susceptible to high-altitude pulmonary edema; HAPE-r = subjects resistant to high-altitude pulmonary edema. OR = odds ratio, 95% CI = 95% confidence interval.

* 1/2 indicated ancestral allele/derived allele according to the NCBI dbSNP database.

† *P* value was calculated by Chi-square test (2*×*2 contingency table).

‡ *P* value was calculated by 2*×*2 contingency table assuming dominant mode (11/12+22) of inheritance on HAPE-s.

§ *P* value was calculated by 2*×*2 contingency table assuming recessive mode (11+12/22) of inheritance on HAPE-s.

|| P = 0.009, OR = 0.28, 95% CI = 0.10–0.76.

# OR = 0.21, 95% CI = 0.08–0.57.

**Figure 1:**
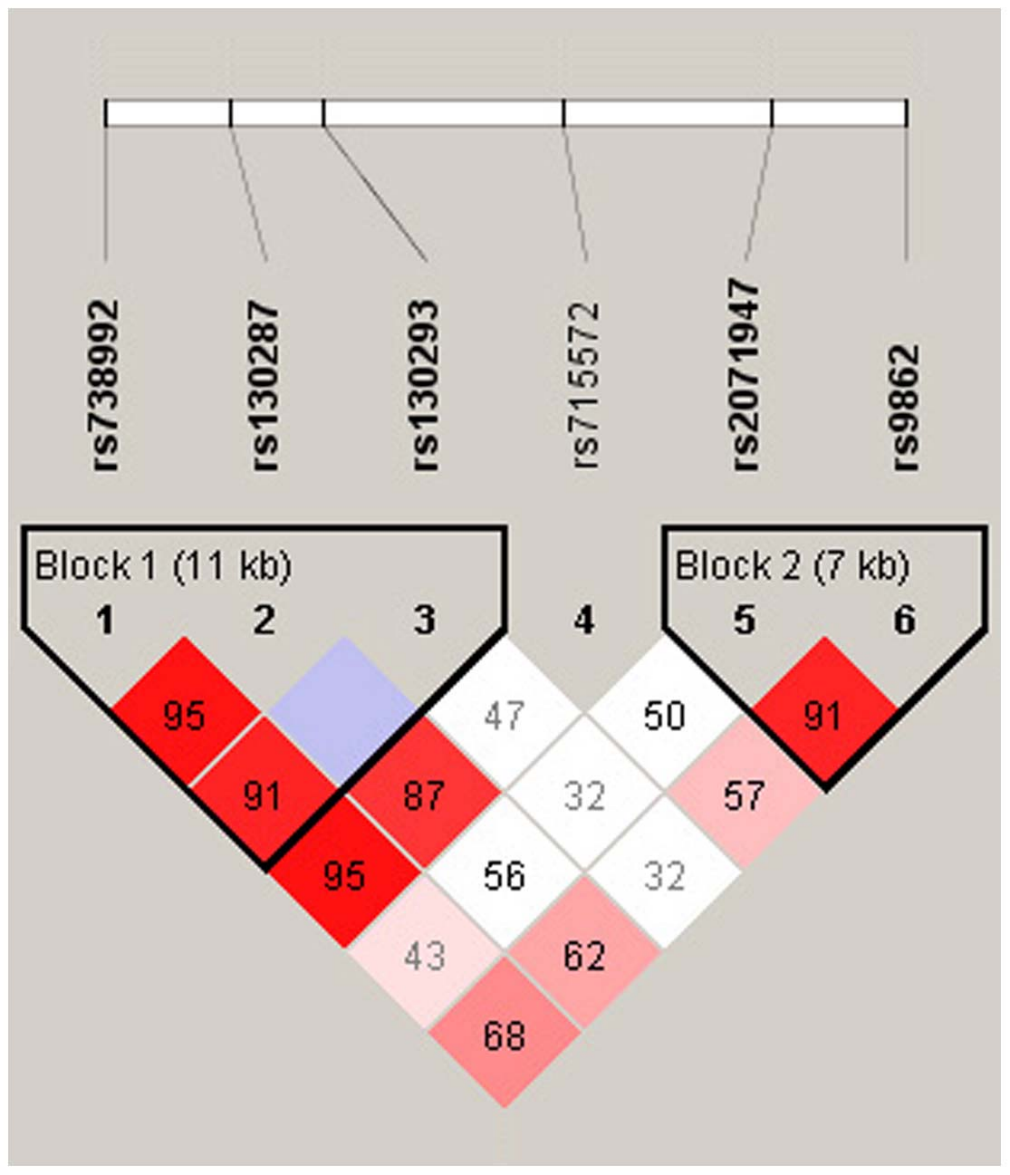
Linkage disequilibrium (LD) plot of six SNPs of the *TIMP3* gene. LD plots were prepared from both subject groups; D’ values that correspond to SNP pairs are expressed as percentages and are shown within the respective squares. Higher D’ values are indicated with a brighter red color. These six SNPs constitute two haplotype blocks that span 11 kb and 7 kb of the *TIMP3* gene.

These six SNPs in *TIMP3* constructed two haplotype blocks in Japanese HAPE-s and HAPE-r subjects according to the D’ values of pair-wise LD (Figure 1). Block 1 was composed of three SNPs (rs738992, rs130287, and rs130293) within a span of 11 kb and block 2 was composed of two SNPs (rs2071947 and rs9862) within a span of 7 kb of the *TIMP3* gene (Figure 1). We identified four common haplotypes with a frequency of more than 0.05 in block 1. The frequency of haplotype CA**C** (constructed by the ancestral allele C of rs738992, ancestral allele A of rs130287, and derived allele C of rs130293) was significantly lower in HAPE-s (0.056) than HAPE-r (0.208, P = 0.0008) subjects with an OR of 0.23 and 95% CI from 0.09 to 0.57, indicating an association of this haplotype with resistance to HAPE (Table 4). There were no significant associations of other haplotypes in block 1 with HAPE-s (Table 4). There were no significant differences in frequencies of the observed haplotypes in the block 2 between the HAPE-s and HAPE-r groups (data not shown).

**Table 4:** The frequencies of the most four common haplotypes from rs738992, rs130287, and rs130293 SNPs in HAPE-s and HAPE-r subjects.

Number of haplotype*	rs738992	rs130287	rs130293	Frequency		*P* ‡
	(C/T)†	(A/G)†	(T/C)†	HAPE-s	HAPE-r	
1	T	A	T	0.514	0.477	0.565
2	C	G	T	0.254	0.224	0.584
3	C	A	C	0.056	0.208	0.0008§
4	C	A	T	0.165	0.083	0.051

Haplotype frequencies were expressed as decimals. A = adenine; C = cytosine; G = guanine; T = thymine; SNPs = single nucleotide polymorphisms; HAPE-s = subjects susceptible to high-altitude pulmonary edema; HAPE-r = subjects resistant to high-altitude pulmonary edema.

* The number of haplotype was defined in this study.

† Ancestral allele/derived allele according to the NCBI dbSNP database.

† *P* value was calculated by Chi-square test (2*×*2 contingency table).

‡ *P* value was calculated by 2*×*2 contingency table.

§ Odds ratio = 0.23 with 95% Confidence Interval from 0.09–0.57.

## Discussion

In this case-control genome-wide association study, we firstly scanned the whole genomes of the HAPE-s and HAPE-r subjects from a Japanese population using 400 genetic microsatellite markers with 10.8 cM resolution. This approach identified five markers associated with susceptibility to HAPE and four markers associated with resistance to HAPE. Moreover, the SNP genotyping study suggested that the derived allele C of rs130293 in the *TIMP3* gene was significantly associated with resistance to HAPE with a recessive effect on the inheritance of the phenotype of HAPE-s. Furthermore, the haplotype carrying the derived allele C of rs130293 was associated with resistance to HAPE. Our results suggested that the prevalent derived allele C of rs130293 might suppress functions of the *TIMP3* gene to protect individuals against HAPE in Japanese populations.

The phenomenon of recurrence of HAPE was firstly reported in Peruvian natives [5], and then in Leadville, Colorado [22]. Vock et al. observed a recurrence rate of 66%, after radiographic evaluation of the chest, in a group of mountaineers with a history of HAPE within one day of climbing to Mt. Capanna Margherita (Monte Rosa, 4,559 m) [6]. Hanaoka et al. reported a 19.6% recurrence of HAPE in 51 Japanese patients [11]. Lorenzo et al. described a family with multiple members, from three generations, affected with HAPE [23]. Candidate genes were hypothesized based on the pathophysiology of endothelial elements in pulmonary circulation in HAPE [24], and genetic polymorphisms in the *NOS3* [9], *ACE* [10], *AGTR1* [10], and *HLA* [11] genes were investigated and found to be associated with susceptibility to HAPE in the Japanese population. However, the present genome scanning did not identify those genes within the 100 kb regions around the significant markers that were supposed to be associated with susceptibility to HAPE in this study. This genome scanning was performed using four hundred microsatellite markers that provided a genome-wide resolution of 10.8 cM. It is estimated that one cM corresponds to about 1 million base pairs in humans on average. Thus, the density of resolution in the present genome scanning was not efficient enough to be able to catch those genetic variants previously reported to be associated with susceptibility to HAPE. It is highly possible that many candidate genes involved with susceptibility to HAPE were undetected in the present genome scanning. Nonetheless, this is the first case-control genome-wide association study aimed at identifying the candidate genes for susceptibility or resistance to HAPE in the field of mountain medicine.

Those genes located within the 100 kb regions around the significant markers (Table 2) seem to be unacquainted with the available knowledge of HAPE according to the gene functions described in NCBI Map Viewer (http://www.ncbi.nlm.nih.gov/mapview/). In addition, the functions of some genes in Table 2 are still unknown or little unknown. Despite of that, gene encoding the heat shock 27 kDa protein family, member 7 (*HSPB7*) and gene encoding chloride channel, voltage-sensitive K (*CLCNK*) within 100 kb of the significant marker D1S2697 (Table 2) potentially appear to be in associations with the susceptibility to HAPE through biological pathways in connection to the available understanding of phenotype of HAPE.^1–4^ Consistently, the SNPs of the heat shock protein 1A and 1B genes (*HSPA1A* and *1B)* were previously reported to be associated with susceptibility to HAPE in a Chinese population [25]. Regarding the function of *CLCNK*, the chloride channel K functions with the sodium (Na+) channel for regulation of cell volume, membrane potential stabilization, signal transduction, and transepithelial transport and plays an important role in salt reabsorption in lungs [26]. Hypoxia inhibited nasal epithelial Na+ transport in both HAPE-s and HAPE-r mountaineers and the activity of the epithelial Na+ channel (ENaC) was lower in the HAPE-s than HAPE-r group [27]–[29], suggesting a contribution of ENaC to the pathophysiology of HAPE. Unfortunately, no information on the genetic variants of the *HSP* and *CLCNK* genes were available from the present HAPE-s and HAPE-r Japanese individuals because there were few observed alleles in the significant marker D1S2697 in our sample size (Table 1). Expanding the simple size and/or upgrading the scanning resolution might lead to a more advanced study in the future.

We paid close attention to the *TIMP3* gene among all the genes within 100 kb of the significant markers listed in the Table 2 according to current information on the role of the *TIMP3* gene in the pathology of lung diseases [21], [30]. *TIMP3* is assigned on chromosome 22 and encoded by 5 exons extending over approximately 55 kb of genomic DNA [31]. The mutations in the exons of the *TIMP3* gene were predicted to disrupt the tertiary structure and, thus, the functional properties of the mature protein [32]. In the present study we found that the derived allele C of the SNP rs130293 (T/C) was significantly associated with resistance to HAPE (OR = 0.22 with 95%CI from 0.09 to 0.55, Table 3) and recessive inheritance of the phenotype of HAPE-s (P = 0.0012, Table 3), the distribution of the T/C heterozygous genotype was more prevalent in the HAPE-r than HAPE-s subjects, and the haplotype carrying the derived C allele was associated with resistance to HAPE. All these results indicated that the derived allele C played a protective role against the susceptibility to HAPE. The SNP rs130293 is located in intron 1 of the *TIMP3* gene. This SNP does not have any direct influence on the conformation of the TIMP3 protein molecule, according to updated biogenetic data. However, this T/C mutation may have an effect on mRNA stability and transcription and/or translation efficiency. This might influence the function of the TIMP3 protein molecule and interfere with its biological properties [33].

TIMPs play a crucial role in the physiological turnover of the extracellular matrix (ECM) by tightly regulating matrix metalloproteinase (MMP) activities [34]. TIMP-3 is the only TIMP that binds tightly to the ECM. The ECM is an extracellular part of the tissues and usually provides structural support to the cells in the interstitial matrix and the basement membrane in addition to performing various other important functions [35]. The balance between MMPs and TIMPs plays an important role in maintaining the integrity of healthy tissues and the disturbance of the TIMP/MMP system is implicated in various pathologic conditions in lungs, including pulmonary inflammation, edema, emphysema, and fibrosis, where loss of ECM integrity is a principal feature [36]. One of the important pathogeneses of HAPE is stress failure of pulmonary capillaries, which generates the high permeability form of edema due to the escape of high molecular weight proteins and blood cells into the alveolar spaces in the lungs of patients [1]. The pulmonary capillaries are composed of a single layer of endothelial cells and supported by the ECM in the interstitial space of lungs. Interstitial pulmonary edema was frequently observed in recreational climbers in high altitudes [37]. We propose that the strength and elasticity of the interstitial space of the lungs might be distinct between HAPE-s and HAPE-r subjects because of the delicate balance in the TIMP/MMP system, determined by the genetic variants involved.

Independent from its MMP-inhibitory activity, TIMP3 encodes a potent angiogenesis inhibitor that inhibits VEGF-mediated angiogenesis by blocking the binding of VEGF to VEGF receptor 2 and inhibiting downstream signaling and angiogenesis [38]. Coincidentally, the level of VEGF in the bronchoalveolar lavage fluid of the patients with HAPE was suggested to play an important role in the repair process for impaired cell layers [39]. Taken together, the *TIMP3* gene is a novel candidate gene for susceptibility to HAPE in the Japanese population.

The major limitation of the present case-control study was the relatively small sample sizes, especially for HAPE-s subjects. In our study, the HAPE-s subjects were strictly selected by differentiating the patients with HAPE from those with acute mountain sickness (AMS) using computer tomography examination, as these two diseases have similar clinical manifestations in the early stages but different pathogeneses. We expect any replication study for the genetic variants in the *TIMP3* gene to have bigger sample sizes to approach the true population values [40]. Nevertheless, the present finding of an association of genetic variants in the *TIMP3* gene with HAPE open a new avenue, the pulmonary interstitial structure, in addition to the endothelial or epithelial components for elucidating the roles of genetic elements in the pathogenesis of HAPE.

In conclusion, this genome-wide association study revealed several novel candidate genes that were associated with susceptibility/resistance to HAPE. Among those, the derived allele C of rs130293 in the *TIMP3* gene might have a resistant role in the susceptibility to HAPE and lead to recessive inheritance of the phenotype of HAPE-s in the Japanese population.
